# Ethnobotanical study of traditional edible plants used by the Naxi people during droughts

**DOI:** 10.1186/s13002-016-0113-z

**Published:** 2016-09-12

**Authors:** Lingling Zhang, Zhenzhen Chai, Yu Zhang, Yanfei Geng, Yuahua Wang

**Affiliations:** 1 South China Botanical Garden, Chinese Academy of Sciences, Guangzhou, 510650 China; 2 Kunming Institute of Botany, Chinese Academy of Sciences, Kunming, 650201 China; 3 University of Chinese Academy of Sciences, Beijing, 100049 China

**Keywords:** Diverse edible plants, Food security, Drought, Naxi, Traditional ecological knowledge

## Abstract

**Background:**

Since 2009, millions of people have been forced to live under food shortage by the continuous drought in Southwestern China. The market was the primary source of aid grains, and fears that the market will be unable to provide sufficient food make safeguarding food security in the face of climate change crucial. Traditional adaptive strategies of pre-market indigenous people are a potential source of innovation. We studied three questions among the Naxi people: 1) What edible plants did they consume during droughts? 2) How did they produce enough food? 3) How did they consume these plants? This study investigates and documents traditional Naxi food knowledge to safeguard food security during drought and facilitate Chinese policy decisions.

**Methods:**

Ethnobotanical investigation was conducted through literature review, semi-structured interviews, collaborative fieldwork and group discussions in three Naxi villages. 89 informants (including 35 key informants) were surveyed from 2012 to 2013. Significant Index (SI) was adopted to evaluate each edible plant’s food supply significance. Voucher specimens were collected for taxonomic identification.

**Results:**

1) In total, 141 edible plants (38 cultivated and 103 wild) were consumed—primarily landrace crops, supplementary edible plants and famine plants. 2) Naxi people produced sufficient food through widespread food production systems, strong landrace crop resilience, and diversity in wild edible plants. 3) Through a diverse diet and consuming almost all edible parts of the plant, the Naxi used edible plants fully to meet food and nutrition needs during drought.

**Conclusions:**

Edible plant diversity is a cornerstone of drought food security. Cultivated crops (especially landrace plants) and wild edible plants were both important. Naxi people protect edible plant diversity through ecological morality and traditional ecological knowledge (TEK). National inventories of edible plant diversity and studies of the TEK of other Chinese indigenous peoples should be undertaken to inform sustainable food policy decisions in China.

## Background

During the last 5 years, southwestern China has suffered from a continuous catastrophic drought, resulting in significant reduction of agricultural production and challenging food security [1]. Yunnan Province was the most affected area [2]. A total of 8.23 million people in the province were affected by food shortages and required aid [3]. People are becoming increasingly dependent on the market for food supplies. During the last 5 years of drought, when we were performing ethnobotanical investigation in indigenous villages, we found that their main source of aid grains was from market. Because global food supply balance is becoming increasingly unstable as the population grows [4], there is uncertainty and fear about the consequences of the market being unable to provide sufficient food.

The drought in China highlights future climate threats [5]. Global and regional weather conditions are becoming more variable under global climate change, with increases in the frequency and severity of extreme events such as droughts, cyclones, floods, and hailstorms [6–8]. By inducing greater fluctuations in crop yields and local food supplies, climate events can adversely affect food production and thus food security [6]. Droughts, which are the primary cause of short-term fluctuations in food production, will become more severe and frequent in semiarid and subhumid areas [6], leading to dramatically reduced crop yields, livestock numbers and productivity [6]. China will suffer more frequently from droughts because global climate change will reduce rainfall in this region. Risks related to crop production and food security are therefore expected to increase [9–11]. China is a large agricultural country with a large population, and Chinese ability to safeguard food security during climate change is a vital issue. It is projected that a wide range of between 5 million and 170 million additional people will be at risk of experiencing hunger by 2080 [6], and there is general consensus that China’s agricultural sector will be significantly affected [12]. In a worst-case scenario, there could be a drop in China’s rainfed yields of rice, wheat, and maize of between 20 and 36 % over the next 20 to 80 years [8]. Safeguarding food security in the context of unfolding changes in weather patterns has been ranked among the top priorities in China’s policy agenda [13, 14], and effective and suitable policies need to be implemented.

Throughout history, communities maintaining strong links to environmental dynamics have developed knowledge, practices, institutions, and beliefs to accommodate recurrent disturbances in securing their livelihoods [15–17]. These cumulatively develop into traditional ecological knowledge (TEK). TEK provides human societies with important resistance and resilience to address disturbances and maintain food supplies under conditions of uncertainty and change [17–19]. TEK contributes to building resistance and resilience in social ecological systems by promoting multiple means of biocultural diversity [20]. Food diversity is a foundation of human society, and the diversity of edible plants, including crops, wild edible plants, fodder, and forage, is a major component of food diversity.

Numerous publications focused on wild edible plants have demonstrated that wild plants are essential components in local diet during both drought and years of adequate rainfall [21, 22]. For example, a study conducted in Zimbabwe revealed that some poor households rely on wild fruits for a quarter of all dry season meals [21, 23]. In Kenya, utilization of indigenous fruits for consumption and sale was found to be higher among low-income earners and contributed to total household food insecurity coping strategies [24]. In KwaZulu-Natal, a study found that Traditional Leafy Vegetables (TLV) have the potential to contribute to household food security by providing direct access to readily accessible nutrients [25]. Ethiopians possess sound knowledge, traditions, and opportunities for using wild plants (including fruits, leafy vegetables, and starchy roots) as supplements to address the problem of often lacking an adequate and constant food supply [26].

In Northwest Yunnan,173 wild edible plant species belonging to 76 families and 139 genera were recorded used by Naxi People in Baidi Village, and *Cardamine macrophylla*, *C. tangutorum* and *Eutrema yunnanense* have traditionally been consumed as an important supplement to the diet, particularly during food shortages as wild vegetables [27]. These studies all suggest that wild edible plants may play a role in moderating food shortage, and specific cultivated crops, especially landrace crops, more or less avoided shortages.

Smallholder cultural landscapes are biocultural refugia that safeguard practices for promoting biodiversity [28]. Agriculture, as practiced in smallholder-farmer dominated landscapes as opposed to large-scale farming, is the backbone of food security in the developing world [28]. Lijiang is listed by UNESCO as a world natural and cultural heritage site, and the Naxi people are the main indigenous group in this area. They are smallholder-farmers practicing a hybrid strategy of planting, farming, grazing, and foraging in the Lijiang mountain area for more than 3000 years [29].

This study was prompted by the continuous drought in southwestern China and the need to seek strategies to safeguard food security other than the market. We investigated and documented traditional knowledge and edible plants used by the Naxi people to safeguard food security in drought before the market economy to assist policy-making in China, using three Naxi villages in Lijiang, northwestern Yunnan, as examples. This study focuses on the following questions: 1) What edible plants did the Naxi People consume? 2) How did they manage to produce enough food to meet demand under drought conditions? 3) How did they consume these plants?

## Methods

### Study site

**Fig. 1:**
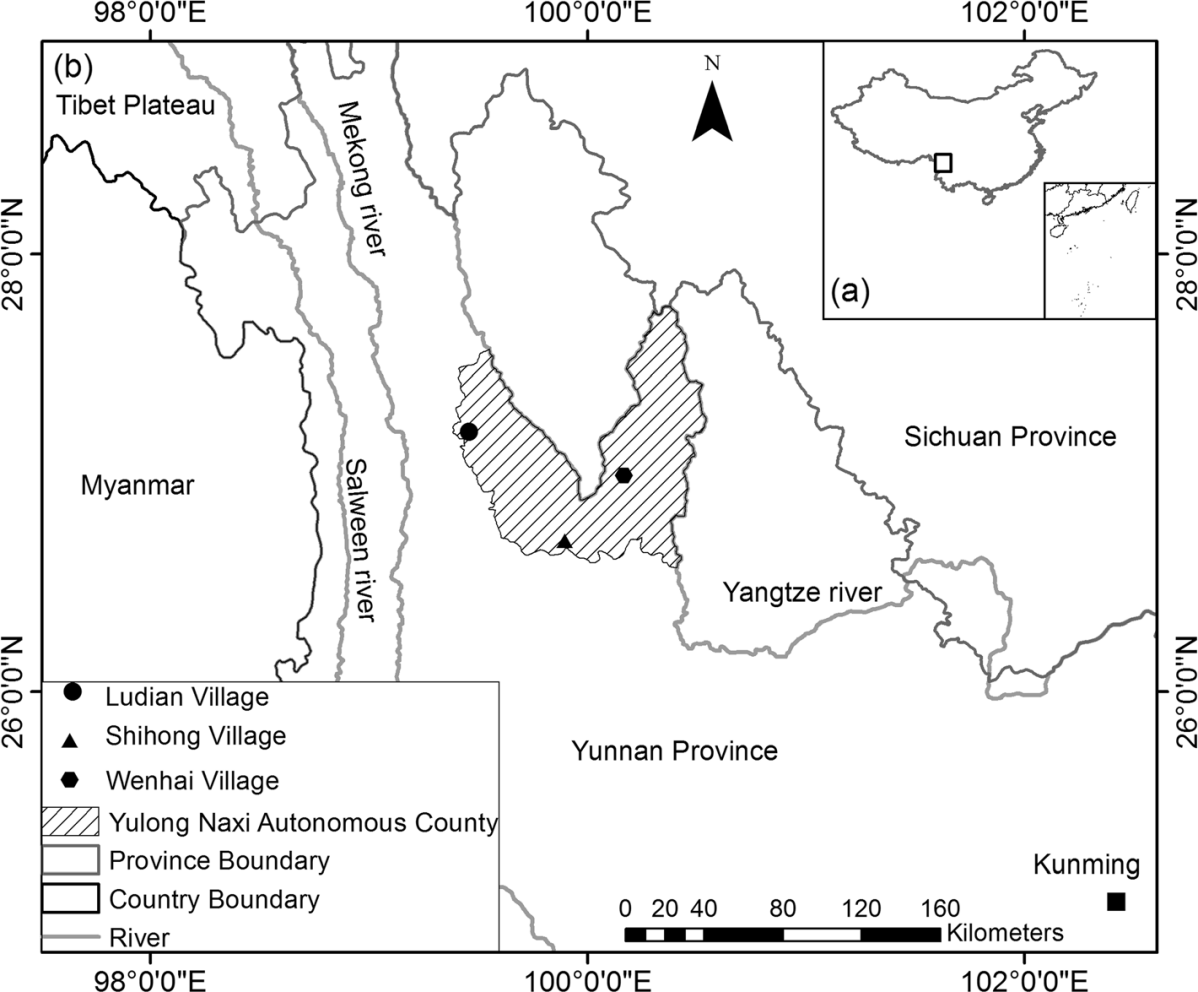
Location of study area in China (a) and investigation villages in the study area (b)

Our case study research focused on the Lijiang area in northwestern Yunnan, which encompasses biocultural refugia and is referred to as the “three valley cultural landscape.” This area is generally representative of smallholder agricultural systems in the surrounding high plateau. Farmland within this cultural landscape is located atop the high-gradient plateau and composed of rainfed fields scattered along the basin and mountain slopes. Three different Naxi villages, Wenhai, Ludian, and Shihong, were selected as the study sites. The criteria for selecting study villages were that they should be smallholder farm villages, that their traditional culture should be well protected, and that they should represent different landscapes relevant to the agricultural patterns in Lijiang (Fig. 1).

Wenhai is a typical wet plateau basin village located directly along the western base of the first peak of the Yulong Snow Mountain, located at 26°59′16_._37″E latitude and 100°10′6.23″N longitude at an altitude of between 3109 and 3380 m. The village is enclosed by high mountains on four sides, and a seasonal lake, the Wenhai Lake, is located in the middle of the plateau. Less than 10 % of the surrounding land is used for agriculture. Wenhai is a very old village and the Ancient Tea Horse Road passes through it on its way to Tibet [30]. Ludian is a typical dry plateau basin village in the central area of the northwestern Yunnan Plateau, located at 27°11′16.54″E latitude and 99°27′36.5″ N longitude at an altitude of between 2400 and 2800 m. Less than 15 % of the surrounding area is used for agriculture. Ludian is the last stop along the “Soul Sending Way,” the transfer road of the Naxi [31]. Shihong is a typical mountain village located along the southwestern portion of the peak of the Laojun Mountain, known as “Ninety-nine Longtan.” The location is at 26°41′24″E latitude and 99°53′49″N longitude, and the altitude ranges between 3500 and 3600 m. Less than 10 % of the surrounding land is used for agriculture. Shihong village was the first to be settled in the area [32]. Its environment and traditional culture are very well preserved.

### Data collection

Our research findings are based on ethnobotanical fieldwork carried out from 2011 to 2013 with the objective of investigating, documenting and interpreting what the Naxi people consumed during drought conditions to safeguard food security, and how they produced and consume those plants. The fieldwork was designed to focus on the three questions articulated in the introduction. A total of 89 households throughout all three villages were selected using the “snowball” technique. Households were selected from among those that still engage in farming as their main activity. Within the households, 35 “elder experts” (male or female) aged over 65 years who had experienced food shortages during times of drought during the past century were invited as our key informants. The key informants lived almost all of their lives within their communities, farming, pasturing, breeding, foraging, and using their knowledge to produce food.

Ethnobotanical semi-structured interviews were used to document how subjects obtained sufficient food to meet nutrition demand during drought disasters in their traditional contexts. All interviews were agreed by informants before conducting. We asked: 1) Which edible plants did you consume? 2) How did you manage to produce sufficient food items? 3) How did you consume these plants? 4) How significant is each plant in providing food during droughts? During subsequent collaborative fieldwork, edible plants used by the Naxi were collected, a quick inventory was carried out, voucher specimens were collected, habitats recorded, and photographs taken. We collected three specimens of each plant. Specimen identification was performed with the help of experts at the Kunming Institute of Botany, Chinese Academy of Sciences. Specimens were stored within the Institute’s herbarium. Finally, group discussions with key informants were organized separately in the three villages. The information obtained from previous studies and the results of our analysis were discussed and verified at these workshops.

### Significant index

Significant Index (SI) [33, 34] was adopted to evaluate the significance of each edible plant in food provision. For each plant consumed, informants were asked how significant the plant was in providing food during droughts on a scale from 1 to 5, with 5 representing greatest significance. SI was assigned mainly according to the quantity and frequency of the plant consumed. During final group discussions, SI values were discussed and verified by informants and the researcher together [35].

## Results

### Edible plants consumed

In total, 139 edible plants (36 cultivated and 103 wild) were consumed by the Naxi people to satisfy food demand during drought. Those plants were mainly landrace crops, supplementary edible plants and famine plants. Table 1 lists the ethnobotanical information for each plant, including the scientific name, Chinese name, habitat, part used, diet type and SI value.

**Table 1:** Inventory of dietary plants used by the Naxi people during drought in Lijiang area, Northwest Yunnan, China (ranked alphabetically by species)

Voucher Code	Scientific name	Chinese Name	Habitat	Part Used	Diet type	SI
NE125	*Aconitum stapfianum* Hand.-Mazz.***	玉龙乌头	W	Root	Healthcare food	0.5
NE132	*Allium beesianum* W. W. Sm.	蓝花韭	W	Leaf	Vegetable, Seasoning	0.5
NE008	*Allium cepa* var. *proliferum* Regel	红葱	G	Whole plant	Vegetable, Seasoning	0.5
NE133	*Allium chrysanthum* Regel	野葱	W	Whole plant	Vegetable, Seasoning	0.5
NE007	*Allium fistulosum* L.	葱	G	Whole plant	Vegetable, Seasoning	0.5
NE027	*Allium hookeri* Thwaites	宽叶韭	W	Leaf	Vegetable, Seasoning	0.5
NE010	*Allium sativum* L.	蒜	G	Bulb	Seasoning, Healthcare food	0.5
NE023	*Allium tuberosum* Rottl. ex Spreng	韭	G	Leaf, Root	Vegetable, Seasoning	0.5
NE139	*Amygdalus persica* L.	桃	V	Fruit	Fruit	0.5
NE102	*Aralia chinensis* L.	楤木	W	Tender stem and leaf	Vegetable	2
NE061	*Arceuthobium pini* Hawksworth et Wiens	高山松寄生	W	Leaf	Drink, Healthcare food	0.1
NE005	*Arctium lappa* L.	牛蒡	W	Root	Vegetable, Healthcare food	1
NE018	*Aruncus sylvester* Kostel.	假升麻	W	Tender stem and leaf	Vegetable	2
NE078	*Athyrium kuratae* Serizawa	仓田蹄盖蕨	W	Tender stem and leaf, Root	Vegetable, Substitute grain, Wine-plant	5
NE062	*Auricularia auricula* (L. ex Hook.) Underw	黑木耳	W	Whole plant	Vegetable, Healthcare food	0.5
NE068	*Avena sativa* L.	燕麦	F	Seed	Grain	5
NE076	*Berberis liophylla* Schneid.	滑叶小檗	W	Flower	Vegetable	0.1
NE082	*Berberis pruinosa* Franch.	粉叶小檗	W	Flower	Vegetable	0.1
NE087	*Berberis wilsonae* Hemsl.	金花小檗	W	Flower	Vegetable	0.1
NE142	*Berchemia sinica* C. K. Schneider	勾儿茶	W	Fruit	Fruit	0.5
NE054	*Brassica chinensis* L.	青菜	G	Aerial part	Vegetable	3
NE071	*Brassica chinensis* L. cv ‘Dakucai’	大苦菜	G	Aerial part	Vegetable	3
NE057	*Brassica juncea* (L.) Czern.	油芥菜	F	Seed	Oil	2
NE024	*Brassica oleracea* var. *capitata* L.	甘蓝	G	Leaf	Vegetable	1
NE032	*Brassica rapa* L.	芜青	F, BF	Fleshy root, Leaf, Tender leaf	Grain, Fruit, Vegetable	5
NE123	*Campylotropis hirtella* (Franch.) Schindl.	毛杭子梢	W	Leaf	Drink	0.1
NE070	*Cannabis sativa* L.	大麻	BF	Seed	Oil, Snack	0.5
NE108	*Capsella bursa*-*pastoris* (L.) Medic.	荠	W	Aerial part	Vegetable, Healthcare food	3
NE046	*Caragana franchetiana* Kom.	云南锦鸡儿	W	Flower	Vegetable	0.5
NE100	*Chaenomeles speciosa* (Sweet) Nakai	皱皮木瓜	V	Fruit	Fruit, Seasoning	1
NE020	*Chenopodium album* L.	藜	W	Aerial part	Vegetable	3
NE021	*Cicer arietinum* L.	鹰嘴豆	F	Seed	Grain, Snack	1
NE112	*Cirsium chlorolepis* Petrak ex Hand.-Mazz.	两面刺	W	Root	Vegetable, Healthcare food	1
NE113	*Cirsium eriophoroides* (Hook. f.) Petrak	贡山蓟	W	Root	Vegetable, Healthcare food	1
NE092	*Cirsium griseum* Levl.	灰蓟	W	Root	Vegetable, Healthcare food	1
NE093	*Cirsium lidjiangense* Petrak ex Hand.-Mazz.	丽江蓟	W	Root	Vegetable, Healthcare food	1
NE037	*Codonopsis convolvulacea* Kurz. var. *pinifolia* (Hand. -Mazz.) Nannf.	松叶鸡蛋参	W	Root	Vegetable, Healthcare food	0.5
NE052	*Coriandrum sativum* L.	芫荽	G	Whole plant	Vegetable, Seasoning	0.5
NE084	*Cotoneaster franchetii* Bois	西南栒子	W	Fruit	Fruit	0.5
NE090	*Cotoneaster microphyllus* Lindl.	小叶栒子	W	Fruit	Fruit	0.5
NE064	*Crataegus chungtienensis* W. W. Smith	中甸山楂	V	Fruit	Fruit, Healthcare food	1
NE063	*Crataegus scabrifolia* (Franch.) Rehd.	云南山楂	V	Fruit	Fruit, Healthcare food	1
NE004	*Cynanchum auriculatum* Royle ex Wight	牛皮消	W	Milky-juice	Coagulating agent	0.1
NE034	*Dioscorea delavayi* Franch.	高山薯蓣	W	Root	Vegetable, Healthcare food	1
NE035	*Dioscorea hemsleyi* Prain et Burkill	粘山药	W	Root	Vegetable, Healthcare food	1
NE045	*Elaeagnus umbellata* Thunb.	牛奶子	W	Fruit	Fruit	0.5
NE127	*Eriophyton wallichii* Benth.	绵参	W	Root	Vegetable, Healthcare food	0.1
NE025	*Fagopyrum esculentum* Moench	荞麦	F, BF	Seed, Tender stem	Grain, Vegetable	4
NE026	*Fagopyrum tataricum* (L.) Gaertn.	苦荞麦	F, BF	Seed, Tender stem	Grain, Vegetable	5
NE051	*Fargesia communis* Yi	马亨箭竹	W	Bamboo shoot	Vegetable	0.1
NE048	*Fargesia melanostachys* (Hand.-Mazz.) Yi	黑穗箭竹	W	Bamboo shoot	Vegetable	0.1
NE050	*Fargesia yulongshanensis* Yi	玉龙山箭竹	W	Bamboo shoot	Vegetable	0.1
NE053	*Fargesia yunnanensis* Hsueh	昆明实心竹	W	Bamboo shoot	Vegetable	0.1
NE001	*Foeniculum vulgare* Mill.	茴香	G	Aerial part, Seed	Vegetable, Seasoning	1
NE095	*Fragaria gracilis* Lozinsk.	纤细草莓	W	Fruit	Fruit	1
NE096	*Fragaria moupinensis* (Franch.) Card.	西南草莓	W	Fruit	Fruit	1
NE094	*Fragaria vesca* L.	野草莓	W	Fruit	Fruit	1
NE011	*Galeopsis bifida* Boenn.	鼬瓣花	W	Seed	Oil	1
NE129	*Galium elegans* Wall. ex Roxb.	小红参	W	Root	Vegetable, Healthcare food	0.5
NE120	*Gentiana cephalantha* Franch. ex Hemsl.	头花龙胆	W	Aerial part	Fermenting agent	0.1
NE116	*Gentiana rigescens* Franch. ex Hemsl.	滇龙胆草	W	Aerial part	Fermenting agent	0.1
NE118	*Gentiana szechenyii* Kanitz	大花龙胆	W	Aerial part	Fermenting agent	0.1
NE101	*Helwingia japonica* (Thunb.) Dietr.	青荚叶	W	Tender leaf	Vegetable	3
NE019	*Hemerocallis citrina* Baroni	黄花菜	G	Flower	Vegetable	0.5
NE022	*Hippolytia delavayi* (Franch. ex、W. W. Smith) Shih	川滇女蒿	W	Aerial part	Vegetable, Healthcare food	0.5
NE009	*Hordeum vulgare* L.	大麦	F, BF	Seed	Grain	3
NE012	*Juglans sigillata* Dode	泡核桃	V	Kernel	Nut	0.5
NE006	*Ligusticum chuanxiong* Hort.	川芎	G	Aerial part, Root	Vegetable, Healthcare food	2
NE141	*Lilium duchartrei* Franch	宝兴百合	G	Root	Vegetable, Healthcare food	0.5
NE143	*Lilium lancifolium* Thunb.	卷丹	G	Root	Vegetable, Healthcare food	0.5
NE028	*Lobaria fuscotomentosa* Yoshim.	黑毛肺衣	W	Whole plant	Substitute grain	5
NE014	*Lobaria orientalis* (Asahina) Yoshim.	东方网肺衣	W	Whole plant	Substitute grain	5
NE002	*Lobaria yunnanensis* Yoshim.	云南网肺衣	W	Whole plant	Substitute grain	5
NE017	*Maianthemum atropurpureum* (Franch.) LaFrankie	高大鹿药	W	Tender stem and leaf	Vegetable	3
NE016	*Maianthemum forrestii* (W. W. Sm.) LaFrankie	抱茎鹿药	W	Tender stem and leaf	Vegetable	3
NE015	*Maianthemum henryi* (Baker) LaFrankie	管花鹿药	W	Tender stem and leaf	Vegetable	3
NE036	*Malus pumila* Mill.	苹果	G	Fruit	Fruit	1
NE091	*Malus rockii* Rehd.	丽江山荆子	W	Fruit	Fruit, Healthcare food	1
NE067	*Malva verticillata* L.	野葵	W	Tender stem and leaf	Vegetable, Healthcare food	1
NE080	*Origanum vulgare* L.	牛至	W	Aerial part	Spice	0.1
NE060	*Phaseolus lunatus* L.	棉豆	F	Seed	Vegetable, Grain	2
NE069	*Phaseolus vulgaris* L.	菜豆	G	Legume	Vegetable	1
NE114	*Pinus armandii* Franch.	华山松	W	Kernel, Tender leaf, Pollen	Nut, Vegetable, Healthcare food	0.1
NE115	*Pinus yunnanensis* Franch.	云南松	W	Kernel, Tender leaf, Pollen	Nut, Vegetable, Healthcare food	1
NE105	*Pisum sativum* L.	豌豆	F	Seed, Tender stem and leaf	Grain, Vegetable, Snack	2
NE097	*Plantago depressa* Willd.	平车前	W	Aerial part	Vegetable, Healthcare food	1
NE099	*Polygonatum cirrhifolium* (Wall.) Royle	卷叶黄精	W	Rhizome	Healthcare food	0.5
NE103	*Prinsepia utilis* Royle	扁核木	W	Kernel, Tender stem	Oil, Vegetable	3
NE077	*Pteridium revolutum* (Bl.) Nakai	毛轴蕨	W	Tender stem and leaf, Root	Vegetable, Substitute grain, Wine-plant	5
NE145	*Pteridium aquilinum* (L.) Kuhn *var. latiusculum* (Desv.) Underw. ex Heller	蕨	W	Tender stem and leaf, Root	Vegetable, Substitute grain, Wine-plant	5
NE135	*Pyracantha angustifolia* (Franch.) Schneid.	窄叶火棘	W	Fruit	Fruit	2
NE134	*Pyracantha fortuneana* (Maxim.) Li	火棘	W	Fruit	Fruit	2
NE031	*Pyrus pyrifolia* (Burm. f.) Nakai	沙梨	G	Fruit	Fruit	1
NE074	*Quercus aquifolioides* Rehd. et Wils.	川滇高山栎	W	Kernel	Grain, Wine-plant	4
NE072	*Quercus guyavaefolia* H. Leveille	帽斗栎	W	Kernel	Grain, Wine-plant	4
NE075	*Quercus monimotricha* Hand.-Mazz.	矮高山栎	W	Kernel	Grain, Wine-plant	4
NE030	*Raphanus sativus* L. var. *raphanistroides* (Makino) Makino	蓝花子	BF	Seed	Oil	3
NE086	*Rhododendron hippophaeoides* Balf.f. & W. W. Sm	灰背杜鹃	W	Flower	Vegetable	0.1
NE085	*Rhododendron yunnanense* Franch.	云南杜鹃	W	Flower	Vegetable, Healthcare food	0.1
NE126	*Ribes glaciale* Wall	冰川茶藨子	W	Fruit	Fruit	0.5
NE130	*Rosa omeiensis* Rolfe	峨眉蔷薇	W	Tender stem	Vegetable	0.1
NE088	*Rosa omeiensis* Rolfe f. *pteracantha* Rehd. et Wils.	扁刺峨眉蔷薇	W	Tender stem	Vegetable	0.1
NE029	*Rosa rugosa* Thunb.	玫瑰	V	Tender stem, Petal	Vegetable, Spice	0.1
NE089	*Rosa sericea* Lindl.	绢毛蔷薇	W	Tender stem	Vegetable	0.1
NE140	*Rosa soulieana* Crep.	川滇蔷薇	W	Tender stem	Vegetable	0.1
NE040	*Roscoea cautleoides* Gagnep.	滇象牙参	W	Root	Vegetable, Healthcare food	0.5
NE041	*Roscoea cautleoides* Gagnep.	早花象牙参	W	Root	Vegetable, Healthcare food	0.5
NE039	*Roscoea tibetica* Bat.	藏象牙参	W	Root	Vegetable, Healthcare food	0.5
NE137	*Rubus aurantiacus* Focke	桔红悬钩子	W	Fruit	Fruit	0.5
NE106	*Rubus biflorus* Buch.-Ham. ex Smith	粉枝莓	W	Fruit	Fruit	1
NE117	*Rubus niveus* Thunb.	红泡刺藤	W	Fruit	Fruit	1
NE119	*Rubus subornatus* Focke	美饰悬钩子	W	Fruit	Fruit	1
NE122	*Rubus trijugus* Focke	三对叶悬钩子	W	Fruit	Fruit	1
NE104	*Sagittaria trifolia* L.	慈菇	W	Root	Vegetable	1
NE079	*Salix balfouriana* Schneid.	白背柳	W	Inflorescence	Substitute grain	5
NE083	*Salix delavayana* Hand.-Mazz.	腹毛柳	W	Inflorescence	Substitute grain	5
NE081	*Salix spodiophylla* Hand.-Mazz.	灰叶柳	W	Inflorescence	Substitute grain	5
NE058	*Sambucus adnata* Wall. ex DC.	血满草	W	Tender stem, Fruit	Vegetable, Fruit	1
NE042	*Sanguisorba filiformis* (Hook. f.) Hand.-Mazz.	矮地榆	W	Root	Vegetable, Healthcare food	1
NE124	*Saussurea leucoma* Diels	羽裂雪兔子	W	Whole plant	Vegetable, Healthcare food	0.1
NE098	*Schisandra rubriflora* (Franch). Rehd. et Wils.	红花五味子	W	Fruit	Fruit, Healthcare food	0.1
NE013	*Scurrula parasitica* L.	红花寄生	W	Fruit	Snack	0.1
NE049	*Solanum tuberosum* L.	洋芋	F, BF	Tuber	Grain, Vegetable	5
NE128	*Stebbinsia umbrella* (Franch.) Lipsch.	肉菊	W	Root	Healthcare food	0.1
NE047	*Sticta nylanderiana* Zahlbr.	平滑牛皮叶	W	Aerial part	Substitute grain	5
NE110	*Taraxacum mongolicum* Hand.-Mazz.	蒲公英	W	Aerial part	Vegetable, Healthcare food	1
NE066	*Thamnolia subuliformis* (Ehrh.) W. L. Culb.	雪地茶	W	Whole plant	Drink	0.1
NE065	*Thamnolia vermicularis* (Sw.) Ach. ex Schaerer	地茶	W	Whole plant	Drink	0.1
NE109	*Thlaspi arvense* L.	菥蓂	W	Aerial part, Seed	Vegetable, Oil	3
NE056	*Triticum aestivum* L.	普通小麦	F	Seed	Grain	2
NE059	*Usnea longissima* Ach.	长松萝	W	Whole plant	Healthcare food	0.1
NE136	*Vaccinium fragile* Franch.	乌鸦果	W	Fruit	Fruit	0.1
NE121	*Viburnum betulifolium* Batal.	桦叶荚蒾	W	Fruit	Fruit	0.5
NE138	*Viburnum cylindricum* Buch. -Ham. ex D. Don	水红木	W	Kernel	Oil	1
NE003	*Vicia faba* L.	蚕豆	F	Seed	Grain	3
NE073	*Vicia sativa* L.	救荒野豌豆	BF	Tender stem	Vegetable	2
NE111	*Zanthoxylum bungeanum* Maxim.	花椒	V	Fruit, Tender stem and leaf	Seasoning, Healthcare food, Vegetable	0.1
NE144	*Zea mays* L.	玉米	F	Seed	Grain	5
NE146	*Cucurbita moschata* (Duch. ex Lam.) Duch. ex Poiret	南瓜	G	Fruit	Vegetable	3

*W* Wild land, *F* Rich Farmland, *G* Home garden, *BF* Cyclic Fallowed Barren Farmland, *V* Inner village

***Aconitum stapfianum Hand.-Mazz (玉龙乌头) is a highly toxic plant because of the presence of aconitine. The preparation process was very strictly controlled to protect against toxic effects. The cleaned fresh aconitum root is stewed with pork fat, but importantly, it must be stewed in boiling water throughout the entire cooking process, as the addition of any cold water can cause toxic effects. The specific cooking process is as follows: when cooking begins, the water must be fully boiling, and then cleaned, fresh aconitum root and pork fat can be added; during the stewing process, when water needs to be added, it must be fully boiling water, and cool water must not be added, or poisoning may occur. Another key point is that, after eating, people must not be exposed to cold wind and should stay in rooms with windows closed for 1 night

#### Landrace crops as staple foods

Landrace crops consumed by the Naxi are their main staple foods. During droughts that are neither too long nor too severe, production of landrace crops is not substantially reduced because they are strongly resistant. Storage methods for staple crops were also traditionally important for withstanding crop reduction in drought stress. As one informant stated:

*“Turnips and potatoes are our main food. We eat them at nearly every meal. Potato is both our grain and vegetable. Turnip is our grain, vegetable, fruit, and pickle. The [turnip] sprouts are our green vegetable in the winter. We cannot live without these two food items. We store a lot of them to avoid crop reduction in bad weather.” (A farmer from Wenhai village, female, 73 years old).*

Storage of grains has been part of the Naxi lifestyle and culture. Their crop storage awareness is very strong and can be detected in their proverbs. These include, for example: *“When the barns are full of grain, you are at ease,”* and *“Take good care of the crops in order to store grain and keep hunger away.”* A large portion of the harvests of landrace crops are preserved by each household to ensure at least 1 year’s supply of food. An old villager in Wenhai village said:

*“We never eat all of our harvests, and must set aside a portion for storage, as this is our habit. We have our special equipment: a grain drying rack. Every household has a tall grain drying rack used for drying grains [oat and barley], vegetables [turnips] and so on. Especially during the production-reducing time of drought, a large amount of the roots and leaves of turnips were hung on the grain drying rack to dry. We hoarded many tons of potatoes and pickled some of our vegetables. Our storage can reduce the risk of food shortage during a year of drought.” (A farmer from Wenhai village, female, 70 years old)*

#### Buffer of supplementary edible plants

**Fig. 2:**
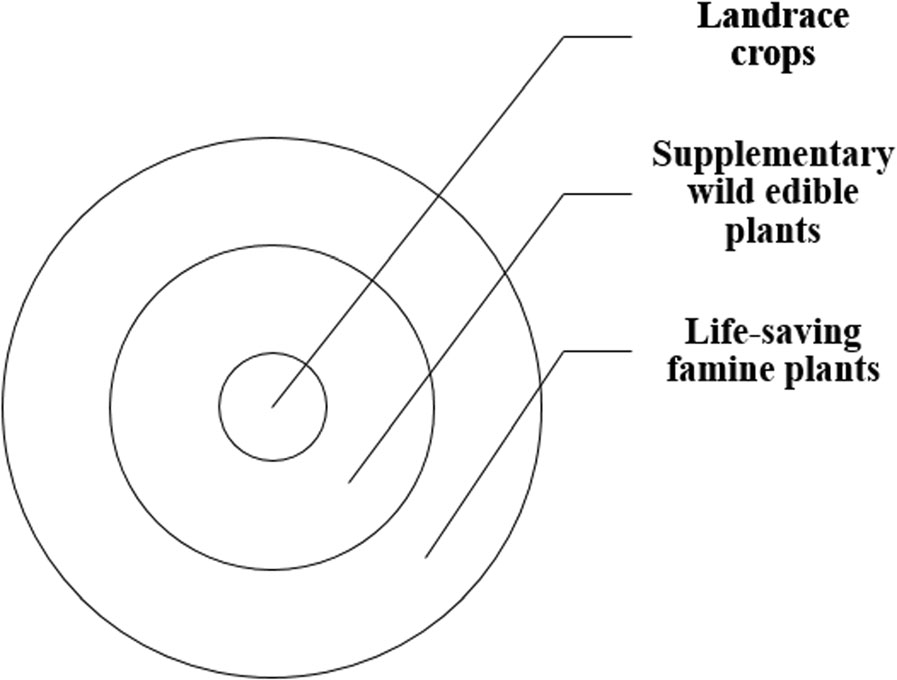
Centrality of landrace crops, supplementary wild edible plants, and life-saving famine plants during drought

The Naxi consume a large number of supplementary edible plants (SI < 0.5). These are mainly wild edible plants. During years when the rainfall and crops are good, these supplementary plants are not frequently used. However, during drought years when the yields of staple edible plants are not sufficient to maintain life, these supplementary edible plants become the main components of the daily diet. Thus, supplementary edible plants can be considered buffers against food shortages during disasters such as droughts (Fig. 2).

#### Life-saving famine plants in extreme drought conditions

Famine plants play a vital role in the survival of individuals and of entire communities during periods of food shortage [16]. Ten main famine plants have been used by the Naxi to avoid starvation during extreme drought periods (Table 2). Villagers in Ludian village said: *“The crops were dried to death, but those plants were able to grow, and though not so tasty, they were enough to avoid starving.”* Among these, bracken was the most important famine plant and was consumed in great quantities during times of food shortage. The buds of brackens grow in early spring (March–April), which is also the time of year when the most severe food shortages occur. Bracken buds can, therefore, help people to overcome famine. As mentioned by respondents:

**Table 2:** The main famine plants and their collection times

Main Famine Plants	Collection Time
*Salix balfouriana*; *Salix spodiophylla*; *Salix delavayana*	Early spring
*Pteridium revolutum*; *Athyrium kuratae*	Early spring
*Lobaria yunnensis*; *Lobaria orientalis*; *Lobaria fuscotomentosa*; *Stica nylanderiana*	Year-round (especially winter)

*“We have great feelings for ferns. During the Ching Ming festival, we use bracken to enshrine our ancestors’ cemeteries. We tell our ancestors that the bracken has grown up, so they no longer have to suffer from hunger. When we are collecting ferns, we usually sing to express our gratitude to the bracken. The song we sing is: ‘Large tracts of bracken have grown out. I come to pick you. You comfort my hungry heart, and I really thank you.’” (A farmer from Wenhai village, male, 70 years old)*

### How to produce sufficient food to meet food requirements

#### Widespread production systems

The Naxi judiciously use nearly all land to produce edible plants. According to their features, main functions, and necessary management, edible plant production systems can be classified as farm systems, including farm land, home gardens, and cyclic fallowed barren farmland, and nonfarm systems, including wild land and the inner village (Table 3).

**Table 3:** Traditional edible plant production systems

Types		Feature	Main Function	Management
Farm System	Farm Land	Limited, average per capita farmland in the three villages is 1.8–3Mu. Not so fertile. Usually on forest edges of mountain foot or in the basin.	Crops growing. Potatoes and turnips are widely planted.	Intensive cultivation
	Home Garden	A greenhouse-like facility close to house. Highly valued. Plant can even grow in winter.	Vegetables cultivating, especially green leafy. Place of introduction and domestication.	Simple facilities agriculture.
	Cyclic Fallowed Barren Farmland	Usually on the slope of mountain. It is barren and not very suitable for cultivation. It is lay fallow after two or three years of farming, after fallow it is farmed aging and this process is circled.	Crops of strong adaptability are planted, mainly Turnips and Tartary Buckwheat. Supplement of farmland.	No management after sowing
Nonfarm systems	Wild Land	Places other than agro-ecosystem and village, including woodlands, grasslands, wetlands and farmland edge.	Numerous wild fruit, vegetable, and famine plants are growing.	No artificial management
	Inner Village	Spare places in village. Frequently human interference.	Many synanthropic plants are growing, especially fruit trees. Place of unconscious introduction.	Frequent disturbance

**Fig. 3:**
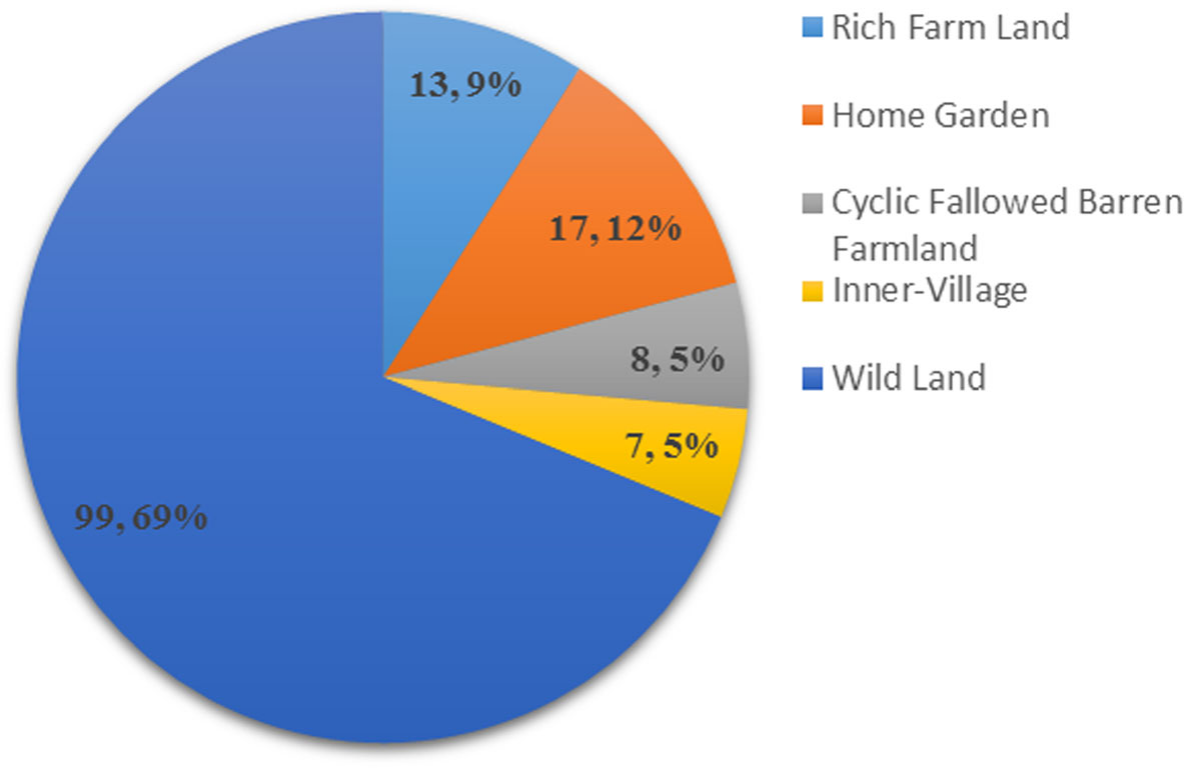
Total species number of traditional edible plants produced in farm and nonfarm systems

**Fig. 4:**
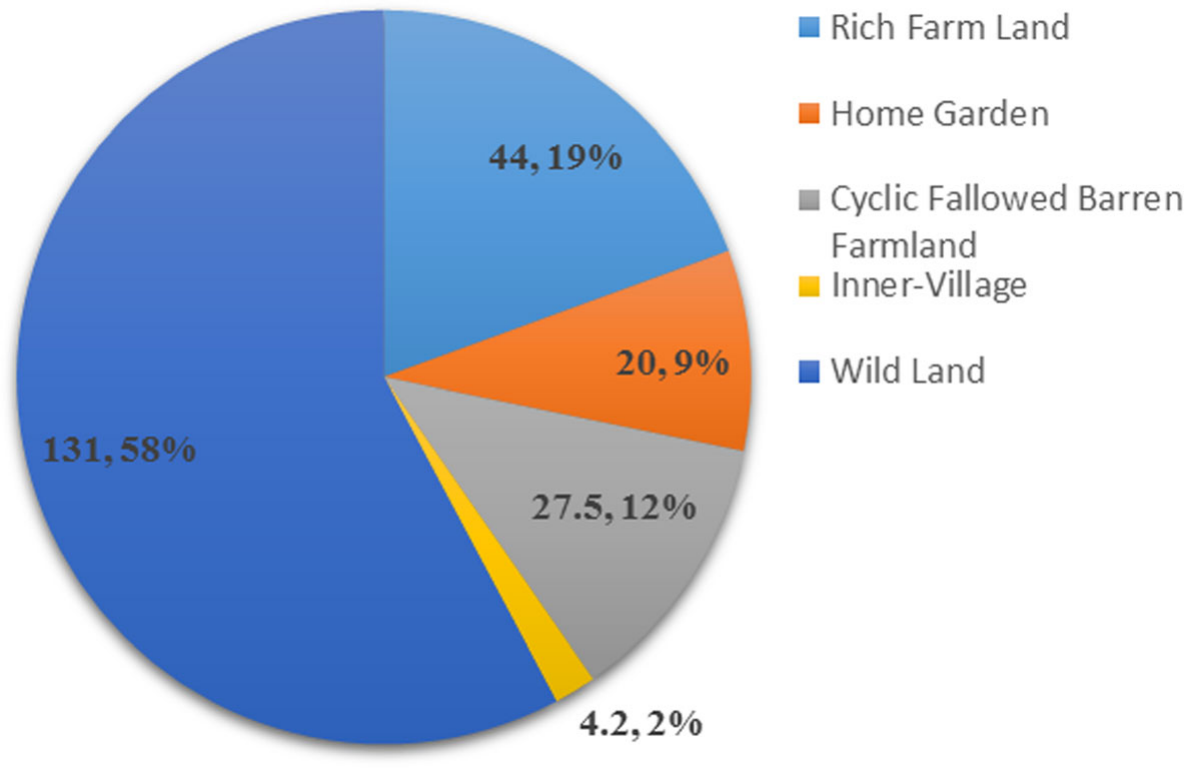
Sum of SI of traditional edible plants produced in farm and nonfarm systems

**Fig. 5:**
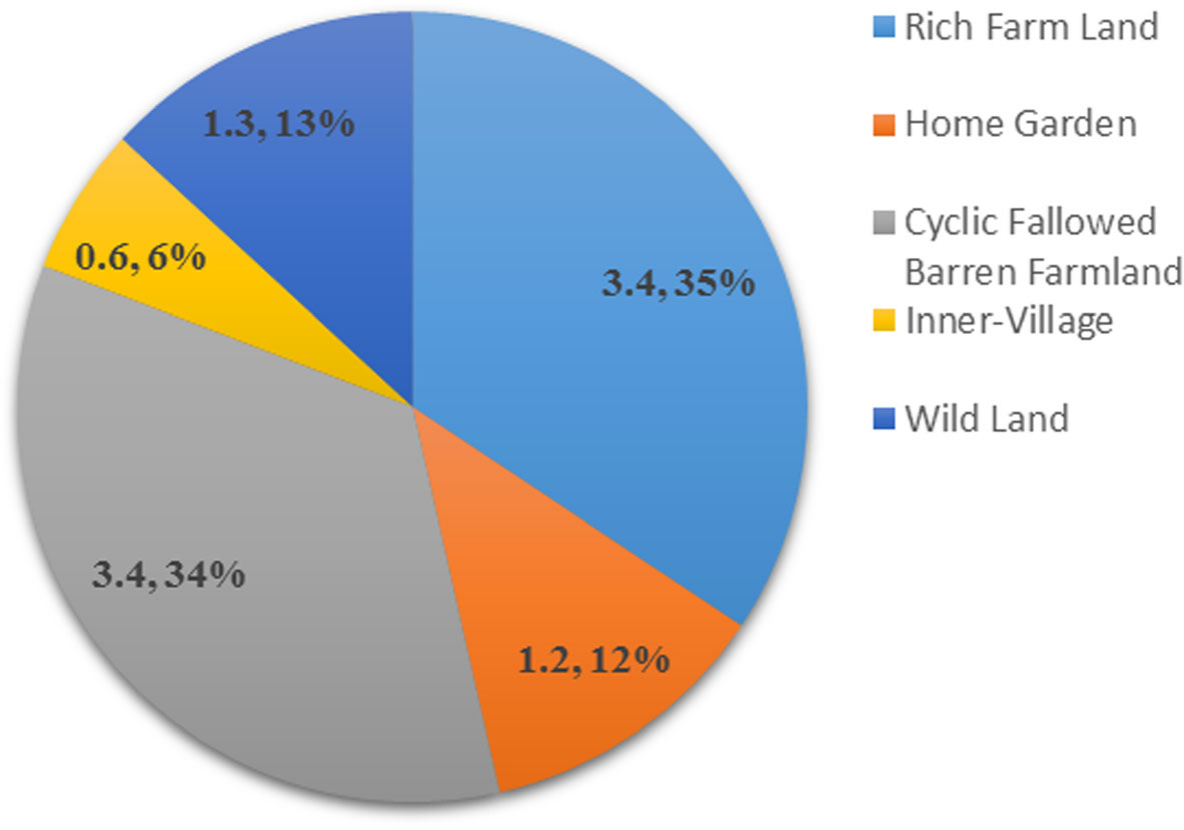
Average SI of traditional edible plants produced in farm and nonfarm systems

**Fig. 6:**
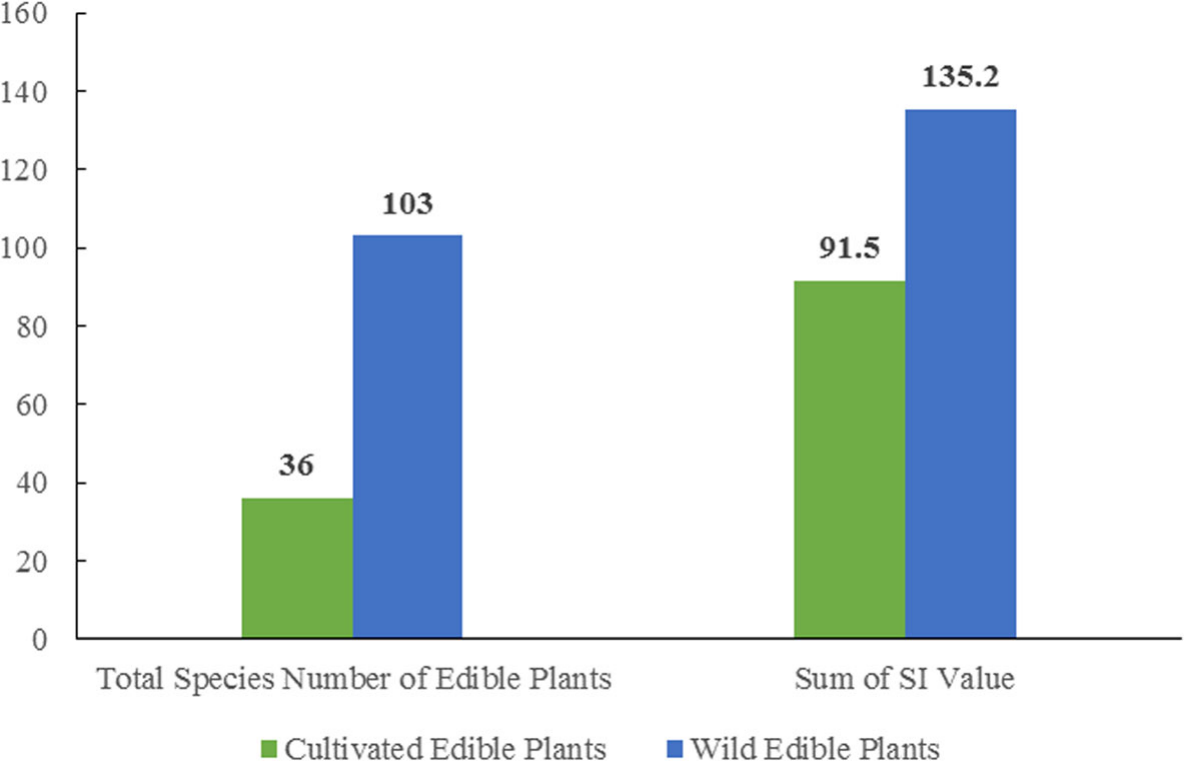
Total species number and sum of SI values of cultivated and wild edible plants

Farm and nonfarm systems all play important roles in edible plant production and food provision. This is evident from total species, sums of SI, and the average SI of the edible plants produced within each system. Figures 3, 4 and 5 show that the nonfarm system provides the largest number of edible plants (106, 76 %) while the farm system plants have the highest average SI (7.99, 81 %) and were consumed more frequently and in greater quantities. Farm and nonfarm systems were both important to satisfy food demand. The gap between the sum of SI values for farm (91.5, 40 %) and nonfarm (135.2, 60 %) system crops is not too large, reflecting the importance of both systems for food supply during drought (Fig. 6). The sum of SI values for nonfarm system is larger which reflect wild edible plants are more important for food supply during drought.

Wild land produced the highest total species (99, 71 %) and SI (131, 58 %) indicating that wild land produces large number of edible plants and was the most important for filling the gap in crop production and food demand during droughts.

#### Diversity of wild edible plants

**Fig. 7:**
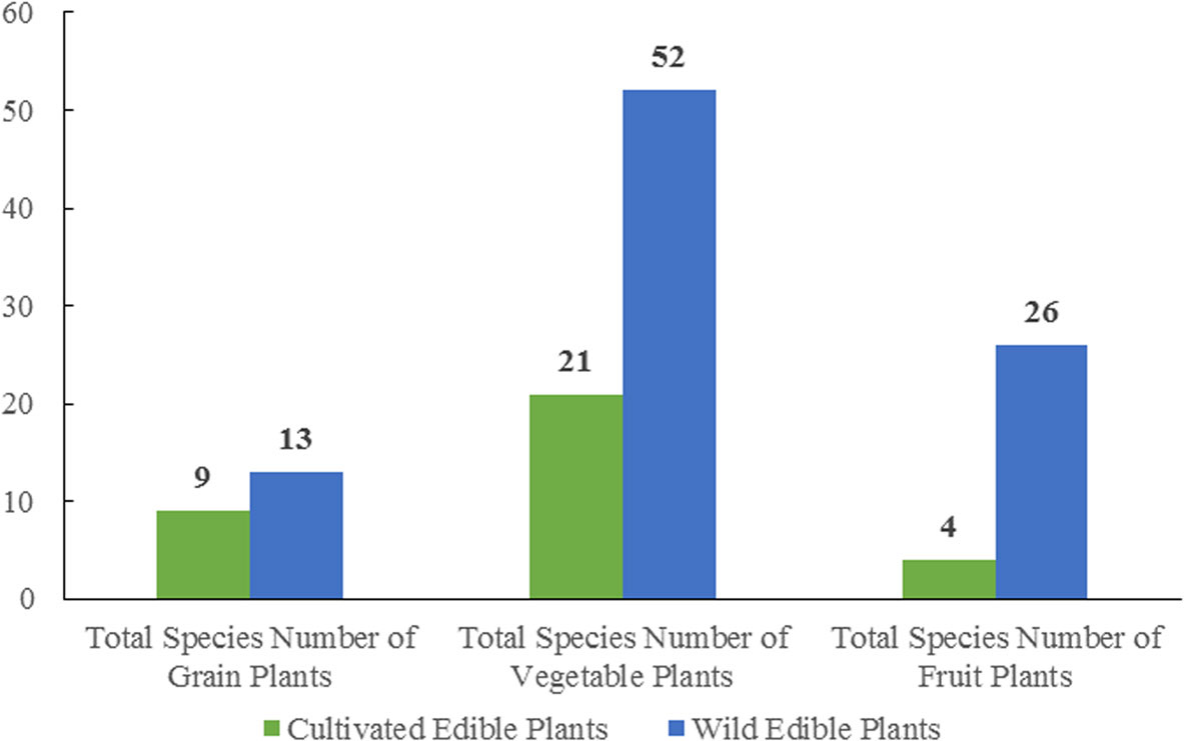
Total species number of cultivated and wild grains, vegetables and fruit plants

**Fig. 8:**
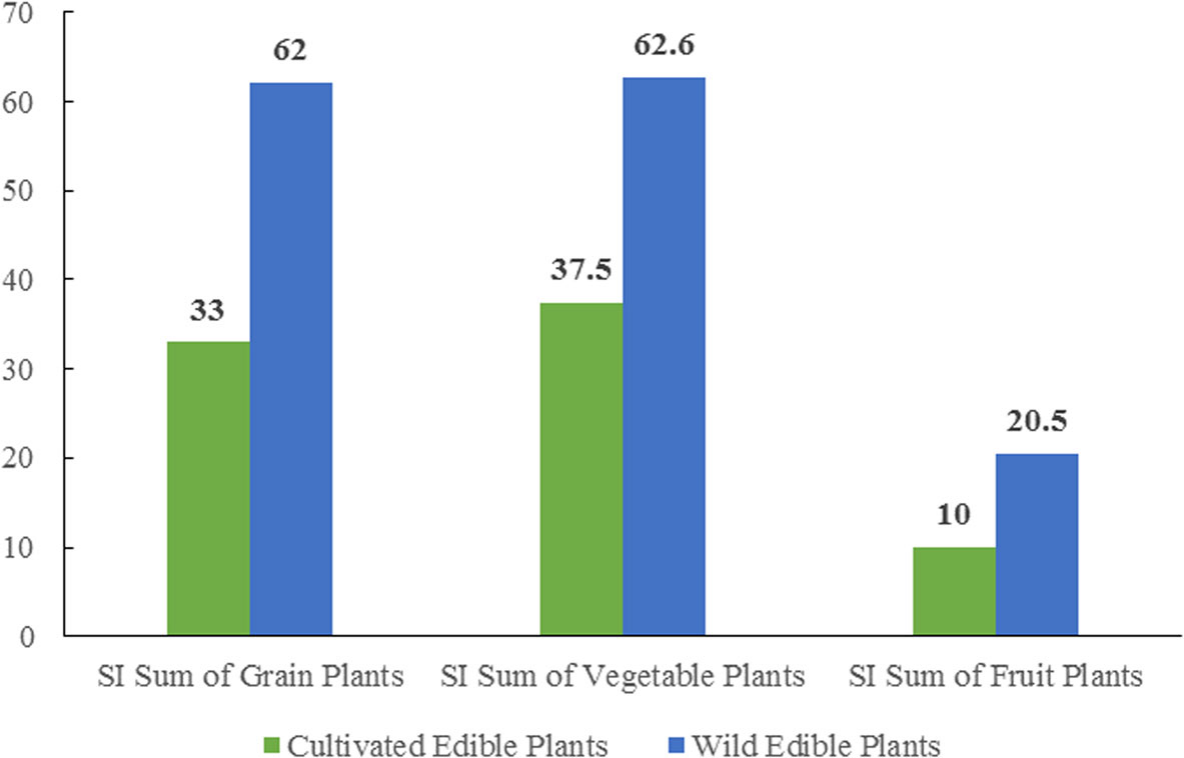
SI sum of cultivated and wild grain, vegetable and fruit plants

The Naxi people consumed 103 species of wild edible plants during droughts, accounting for 74 % of the total plants consumed. Wild edible plants were very important for food provision. Figure 6 shows that total number of wild edible plants (103) was more than three times the number of cultivated plants (36), and the sum of their SI value (135.2) was also much higher than that of the cultivated plants (91.5). Wild edible plants were also very important for nutrition. Figures 7 and 8 show that wild edible plants provided more grains, vegetables and fruits than cultivated plants and were consequently the main sources of required nutrition.

#### Strong resilience of landrace crops

Landrace crops are the cornerstone of local farming and are crucial for lessening the risk of harvest loss attributable to climate fluctuations (and particularly drought) because they endure soil moisture depletion and reduced growing seasons [28]. The landrace crops of the Naxi in Lijiang are potatoes, turnips, and tartary buckwheat, which are all drought-resistant plants. Tartary buckwheat is the best-known fast growing crop, and it adapts well to shortened growing seasons during droughts. Villagers in Shihong village stated the following:

*“The potato and turnip yields can be reduced by drought, but the harvest rarely totally fails. Potato and turnip can get through drought and survive when the rain comes. During a drought period, many pests will accrue; they will eat up the sprouts of seed crops, but potato and turnip roots can escape and they can grow again once the rain has fallen.”*

*“Tartary buckwheat sprouts in less than 3 days, and matures in less than 3 months. We grow buckwheat once the rain comes. Buckwheat grows fast, and we can harvest it before the winter comes.” (A farmer from Shihong village, female, 67 years old)*

### How edible plants were consumed

The Naxi consumed their edible plants fully to meet food and nutrition needs during drought. This was achieved through diverse diet type and consuming almost all edible parts of the plants.

#### Diversity of diet type

**Fig. 9:**
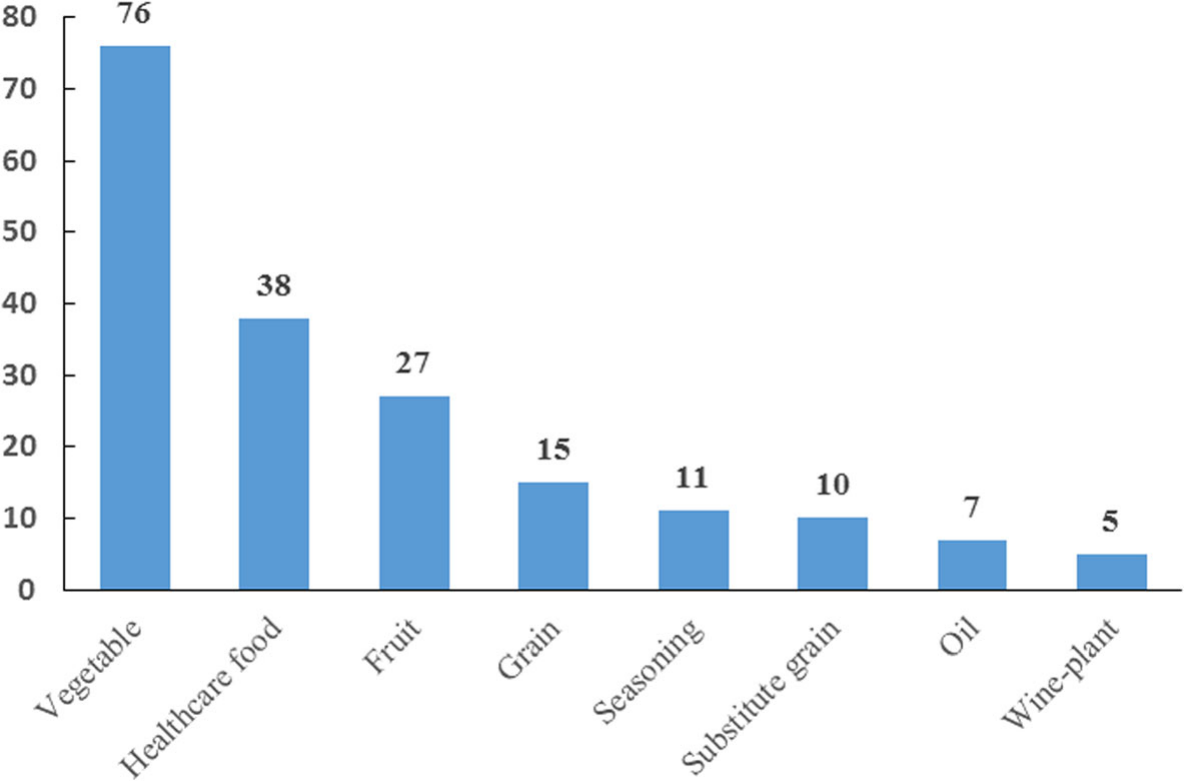
Eight main diet types of traditional edible plants used by the Naxi people in drought

The diet types of traditional edible plants consumed by the Naxi in drought are highly diverse. There are 15 diet types: the most common eight are shown in Fig. 9. The remaining, less common dietary usages were: drinks (4), snacks (4), fermenting agent (3), nut (3), coagulating agent (1) and spice (1). Up to 74 traditional edible plants were consumed in more than one diet type, especially landrace crops such as turnips, potatoes, and bracken (Table S1).

#### Used parts

**Fig. 10:**
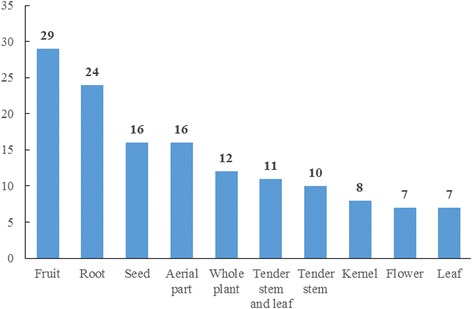
Ten parts of traditional edible plants most frequently used by the Naxi people in drought

The edible parts of traditional plants used by the Naxi people are also highly diverse. A wide range of plant parts are used, including fruits, roots, seeds, aerial parts, whole plants, tender stems and leaves, tender stems without leaves, kernels, flowers, and leaves. The most common 10 plant parts are shown in Fig. 10. The remaining less commonly used parts are bamboo shoots (4), inflorescences (3), tender leaves (3), pollen (2), bulbs (1), fleshy roots (1), legumes (1), milky juice (1), petals (1), rhizomes (1), and tubers (1). Our research revealed that all plant organs (roots, stems, leaves, flowers, fruit and seeds) and parts of plants at different growth stages (buds, tender stems and leaves) could be collected as food in the Naxi village.

## Discussion

Edible plant diversity is the cornerstone of traditional food security in drought. Diversity is present in three levels: production system, species and use. Edible plant diversity safeguard food security in at least two ways: 1) They provide at least basic food needs during droughts. Landrace crops can resist a degree of drought, and when harvest of landrace crops is reduced, supplementary plants can play an important role in food provision. During the most serious droughts, famine plants can be eaten to facilitate survival. 2) Edible plant diversity enhances the resistance and resilience of the food supply system. Some edible plants cannot grow with reduced rainfall, but others can. Drought can reduce production of crops and the biomass of wild edible plants, but diversity allows people to increase variety to offset the reduction, thus enhancing resilience. When drought ends and rain comes, potatoes and turnips can recover quickly, tartary buckwheat can be planted, and within less than 3 months the seeds can be harvested. Wild edible plants including wild vegetable can grow quickly, increasing food system resilience.

Landrace crops and wild edible plants are all the backbone of edible plant diversity in drought time, and both play a role in food provision. The importance of wild edible plants can be seen by comparison with years of adequate rainfall, especially, it can been seen in the most severe food shortage devastate during 1958–1961 [36]. In a normal year, the Naxi people in the three villages mainly eat their staple foods: potatoes, turnips and buckwheat produced in farmland. But during droughts, wild edible plants were largely eaten as supplementary and famine plants. Informants said people in other surrounding villages suffered more than themselves during droughts because they have less forest to provide wild edible items. The severe food shortage of 1958–1961 proved the essential lifesaving role of wild edible plants. For many reasons, people across China suffered from extreme famine, and many people starved to death, during those 3 years [36]; the Naxi people of the Northwest Yunnan were no exception. All the informants we interviewed in the three villages had experienced the disaster. They said they had survived by eating many wild edible plants (i.e., the famine plants in Table 2); especially, brackens were consumed to save lives. When mentioning this history, the villagers were all very proud that no people in their villages starved to death in the 3 years of devastation, because they could collect wild edible plants in their forest (This was the same as Heihe Valley in the Qinling Mountains of central China [37]) and eat them in larger quantities than many people in nearby towns, who starved to death because they had less forest from which to collect wild edible plants. The number of older women who had married from the nearest town into the three villages is suggestive, because the three villages all have large areas of forest that can produce diverse wild edible plants. Women marrying into these villages believed that they and their descendants would not starve to death when drought and other disaster came. The history of 1958–1961 fully supported that wild edible plants were crucial in resisting serious food shortage-related devastation and the crucial role of the forest in food production.

The wild edible plants used by the Naxi people were well prepared to ensure that they were nontoxic and to improve their taste. No health problems were caused when consuming wild edible plants during the drought time. Before cooking, many wild edible plants were boiled for at least 10 min, then put into clean, cool water and soaked for at least half a day, during which time the soaking water was changed more than twice. For special edible plants, the processing time was prolonged. Brackens should be soaked for 2 days, and the water should be changed twice daily. Wild edible plants were usually not eaten alone; they were eaten by adding a small amount of corn flour, tartary buckwheat, or oats. For example, inflorescence of *Salix* was first boiled, soaked, and chopped, and then a small amount of corn flour was added; after that, the material was baked as pancakes to eat. In the few extreme conditions of having only wild edible plants to eat, the taste was not good, but no health problems were caused.

*Aconitum stapfianum* Hand.-Mazz (玉龙乌头) is a highly toxic plant because of the presence of aconitine; it is responsible for many reports of food poisoning-related death [38]. Our survey found that the preparation process was very strictly controlled to protect against toxic effects. The cleaned fresh aconitum root is stewed with pork fat, but importantly, it must be stewed in boiling water throughout the entire cooking process, as the addition of any cold water can cause toxic effects. The specific cooking process is as follows: when cooking begins, the water must be fully boiling, and then cleaned, fresh aconitum root and pork fat can be added; during the stewing process, when water needs to be added, it must be fully boiling water, and cool water must not be added, or poisoning may occur. Another key point is that, after eating, people must not be exposed to cold wind and should stay in rooms with windows closed for 1 night. In Qinling Mountain, preparations of aconitum are also strictly controlled to protect against toxic effects [38], but the process differs between the two areas.

But how do the Naxi people safeguard their edible plant diversity, and how do they protect the ecosystems around their villages that produce large numbers of wild edible plants? Previous studies of the Naxi [32] and our investigation both suggest this is the result of their ecological morality and TEK. The Naxi possesses an “ethic of close relatives” philosophy grounding the relationship between humans and nature [39]. They refer to nature as “Shu” and believe that Shu is the “half-brother” and equal of humans [39]. The Naxi feel indebted to nature when they receive support from it, and they are obliged to pay off these debts [39, 40]. Ecological morality is therefore firmly established in the hearts and minds of all villagers, and they treat this as their code of conduct [37]. Consequently, Naxi people have devised highly effective management strategies, specialized organizations, and strict rules for forest management and preservation [31, 32]. Forests are classified into different usage categories according to their characteristics: pine needles collection forests (pine needles were collected to make organic fertilizer), firewood forests, water conservation forests, grazing forests, timber forests, and Kamiyama. Different forest types require different management methods [32]. Furthermore, there are special organizations and village rules to protect the forests. Each of the three villages has an organization known as “the association of respectable old men” that is responsible for prohibiting forest destruction. Village rules clearly prescribe the number of trees that can be cut for specific purposes. The villages’ forests are well managed and preserved. Rather than being overexploited as farmland, their habitats’ landscapes and ecosystems are reasonably used according to their features to produce diverse edible plants. Ecological morality and TEK preserve the environment’s sustainable productivity and resistance.

Besides preserving forest and wild edible plants, the Naxi also placed great emphasis on their farmland. Their farmland stewardship places high value on soil moisture conservation to improve crop yield. The Naxi’s traditional farming mode is designed to conserve soil moisture; no water is needed when applying fertilizer. Organic manure made from pine leaves is used to improve the soil’s water-holding capacity. Our study showed that every year, each household collected at least 1,000 kg of pine leaves to make manure, and that this collection work was their main labor during the winter.

Obtaining food or learning about edible plants from other ethnicities settled in the area was another way of relieving food shortages during drought years. The Naxi managed to obtain food items to broaden their food diversity by borrowing and exchanging food with the Bai people, who mostly settled in the valley, where the altitude is low, and the land (e.g., that adjacent to the Jingsha River) could still be irrigated. In the Lijiang area, many Naxi people lived together with the Yi people and could learn about wild edible plants from the Yi people. During our investigation, one informant said that the Yi people generally ate more wild edible plants than themselves, because 30–40 years ago, the Yi people lived more on grazing and less on agriculture; thus, they knew much about wild edible plants. For example, the informant said that the Yi people ate Laolongpi (*Lobaria fuscotomentosa*, *Lobaria orientalists*, *Lobaria yunnanensis*) in normal years, but that the Naxi people did not; however, during the years of food shortage, they learned to eat Laolongpi from the Yi people to buffer the food shortage. Exchanges and contacts with other ethnicities living in the area were also important for the Naxi people to improve their edible plant diversity and promote adaptability.

Ecological morality, TEK, and the edible plant diversity of the Naxi people are drastically declining in the context of global change and China’s rapid development, and their food resources are becoming increasingly dependent on the market. In Northwest Yunnan, the use of wild edible plants and related knowledge are eroding rapidly, especially in areas with convenient transportation and booming tourism [41]. They are abandoning their traditional agricultural practices and have forgotten much of their knowledge about the use of traditional edible plants. Promoted by some companies, Maca has become one of the Naxi’s most extensively planted crops, with their planting areas of potatoes and turnips being reduced every year. Many traditional crops, such as *Hordeum vulgare*, *Avena sativa*, and *Raphanus sativus*, are no longer cultivated at all. Large areas of farmland have been abandoned, because the people who would have otherwise cultivated them have become migrant workers in cities. Collecting wild edible plants is now very rare, and the younger generation has discarded the knowledge and use of traditional wild edible plants. Many Naxi people have been educated in schools from a very young age; consequently, they are not familiar with their traditional culture and can barely speak the Naxi language. This situation was common throughout the three villages we studied. A large proportion of their food is purchased from markets. Their traditional lifestyle is disappearing, and their livelihoods are largely controlled by markets.

If markets do not provide sufficient food, and if humans lose their knowledge and ability to produce enough food, what will happen? Our environment is changing, and food security is becoming a major challenge; thus, promoting the ability of human beings to safeguard food security in an uncertain future is very important. Edible plant diversity, including crops and wild edible plants and their associated traditional knowledge, should be considered for any safeguarding strategy. In China, a number of practices have been adopted by the government and farmers to adapt to climate change, and many relevant policies have been designed. It would be wise to pay attention to the diversity of edible plants once used by the country’s many indigenous people and the associated traditional knowledge. We recommend that national legislation be adopted to develop national inventories of traditional edible plants and associated knowledge, especially those once used in times of food shortage, such as edible plants used in times of drought.

## Conclusion

As a large agricultural country with a large population, safeguarding food security under climate change is a challenge for China. China is part of the world market and depends heavily on the market for food supply, even for its indigenous communities, but fear of market failure prompts the search for locally sustainable alternatives to ensure food security during scarcity and drought. China has a long history of traditional agriculture including many smallholder farmers like the Naxi who have accumulated high edible plant diversity and rich TEK to withstand risks. Edible plant diversity is the cornerstone of food security in drought, ensuring basic food needs are met and enhancing the resistance and resilience of the food supply system. Traditional communities are a source of valuable empirical knowledge for safeguarding food security and maintaining diversity. It would be beneficial for China to draw on this experience and knowledge to make policy decisions that are suitable for national conditions. The ecological morality of the Naxi people is a good reference for China to protect its environment while increasing food production, and ecological management strategies, specialized organizations, and strict rules of environmental protection can be made following the Naxi model. More pressingly, the national legislature should immediately begin to develop national inventories of edible plant diversity and to document TEK for protecting this diversity.

## Abbreviations

SI: Significant index
TEK: Traditional ecological knowledge
